# Electrical excitability of cancer cells—CELEX model updated

**DOI:** 10.1007/s10555-024-10195-6

**Published:** 2024-07-08

**Authors:** Mustafa B. A. Djamgoz

**Affiliations:** Department of Life Sciences, Imperial College London, South Kensington Campus, London, SW7 2AZ UK

**Keywords:** Metastasis, Sodium channel, Potassium channel, Action potential, Electrodiagnosis, Electroceutical

## Abstract

The normal functioning of every cell in the body depends on its bioelectric properties and many diseases are caused by genetic and/or epigenetic dysregulation of the underlying ion channels. Metastasis, the main cause of death from cancer, is a complex multi-stage process in which cells break away from a primary tumour, invade the surrounding tissues, enter the circulation by encountering a blood vessel and spread around the body, ultimately lodging in distant organs and reproliferating to form secondary tumours leading to devastating organ failure. Such cellular behaviours are well known to involve ion channels. The CELEX model offers a novel insight to metastasis where it is the electrical excitation of the cancer cells that is responsible for their aggressive and invasive behaviour. In turn, the hyperexcitability is underpinned by concomitant upregulation of functional voltage-gated sodium channels and downregulation of voltage-gated potassium channels. Here, we update the *in vitro* and *in vivo* evidence in favour of the CELEX model for carcinomas. The results are unequivocal for the sodium channel. The potassium channel arm is also broadly supported by existing evidence although these data are complicated by the impact of the channels on the membrane potential and consequent secondary effects. Finally, consistent with the CELEX model, we show (i) that carcinomas are indeed electrically excitable and capable of generating action potentials and (ii) that combination of a sodium channel inhibitor and a potassium channel opener can produce a strong, additive anti-invasive effect. We discuss the possible clinical implications of the CELEX model in managing cancer.

## Introduction

Every cell in the body has an electrical make up, especially a voltage difference across the plasma membrane, called the “membrane potential” (*V*_m_). This enables the cell both to self-function and to communicate with other cells. Although only several tens of millivolts in absolute value, *V*_m_ is equivalent to a voltage gradient of some 10^7^ V/m. This is a huge force that can impact every protein in the cell membrane. Thus, changes in the magnitude of *V*_m_ can have profound effects on functioning of cells, including cancer cells, and cellular networks [1–3]. For example, even just a hyperpolarization of *V*_m_ can bring about differentiation of stem cells, including cancer stem cells [4, 5]. Increasing evidence suggests that cellular bioelectricity generally and *V*_m_ particularly play a significant role in the pathophysiology of cancer e.g. [3, 6–8].

**Fig. 1:**
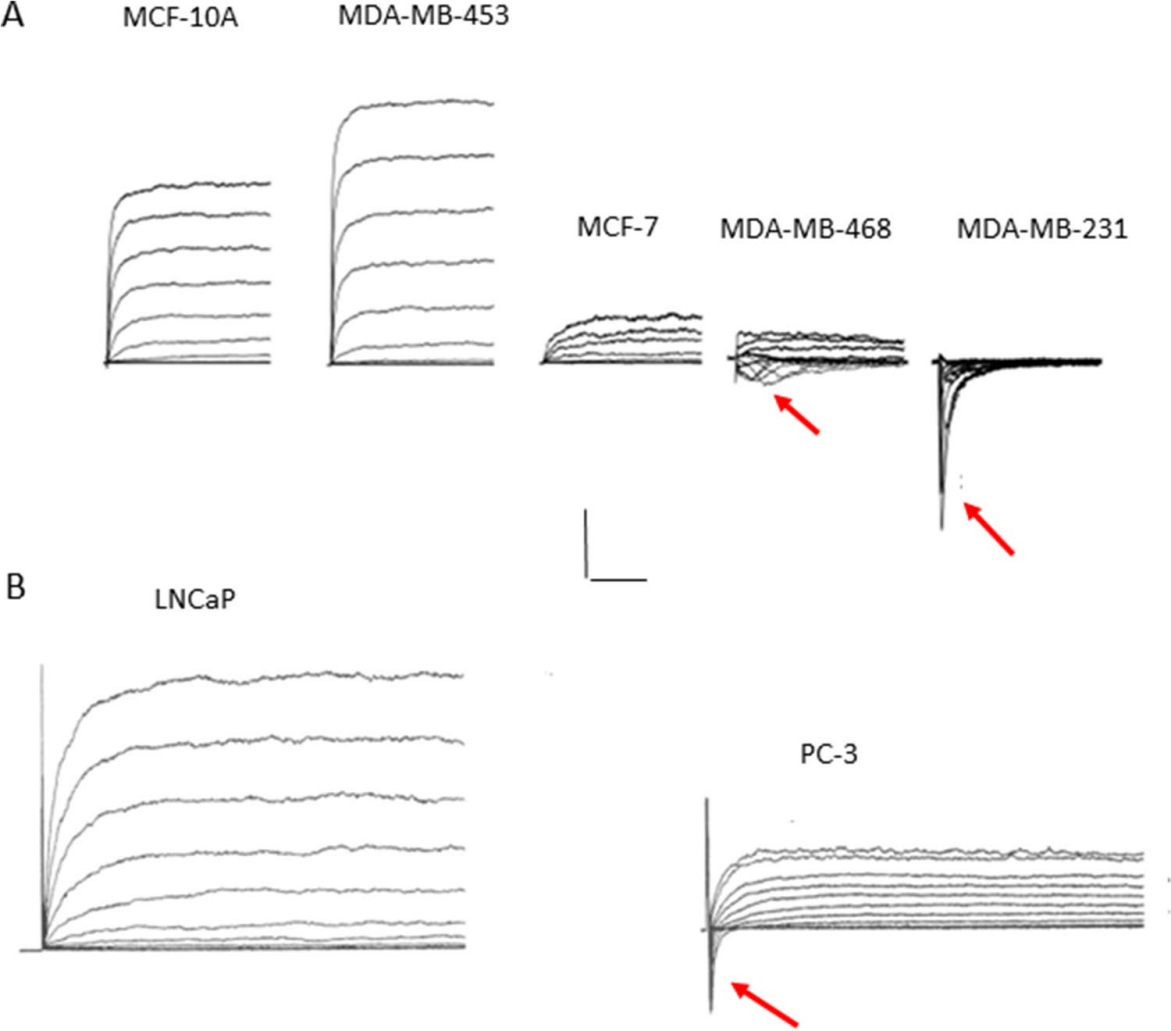
Voltage-activated membrane currents recorded from cells of human breast and prostate cancer cells of markedly different metastatic potential.** A** Five different human breast epithelial cell lines of varying metastatic potential (increasing from left to right). MCF-10A cells are normal cells whilst MDA-MB-231 is the most metastatic. The currents were generated by pulsing the membrane potential from a holding voltage of − 100 mV, in 5 mV steps, from − 60 to + 60 mV for 200 ms. Scale bars: 2 nA/50 ms (MCF-10A and MCF-7), 200 pA/2 ms (MDA-MB-468), 200 pA/10 ms (MD-MB-231). Voltage pulses were applied with a repeat interval of 20 s. For clarity, every second current trace is displayed. **A** Modified from Fraser et al. [11]; extra data provided by Dr. S. P. Fraser. **B** Similar to **A**, data obtained from human prostate cancer cells—strongly metastatic PC-3 and weakly/non-metastatic LNCaP. Scale bars: 1 nA/50 ms. Modified from Laniado et al. [14]. For both carcinomas, increased metastatic potential is associated with decreased amplitude of the cells’ outward currents (note the difference in the scale of the currents in the different cells). Inward VGSC currents (indicated by small red arrows) appear only in the metastatic cells

Here, we evaluate the concept and update the evidence for the “electrical excitability” of cancer cells, formalised originally as the “CELEX” model (celex = cellular excitability) [9, 10]. This was proposed initially from systematic profiling of basic voltage-gated ion channel activity in carcinoma cells of varying metastatic potential [9–11]. In essence, as their metastatic potential increased, the cells’ outward currents, due mainly to voltage-gated potassium channel (VGPC) activity, *decreased*, i.e. the cells appeared to lose what could be their “inhibitory” signals. In parallel, functional voltage-gated sodium channel (VGSC) upregulation (in fact, *de novo* expression) appeared specifically in cells capable of invasiveness/metastasis. This profile is illustrated for human breast epithelial and prostate cancer cells in Fig. 1. Similar profiles have been shown for rat prostate cancer and human non-small lung cancer cells [12, 13]. Such a combination of functional VGSC and VGPC expression would make the membranes of these cells potentially electrically excitable. In turn, it has been proposed that it is this state of excitation that makes the cancer cells hyperactive and aggressive, leading to disruption and invasion of the surrounding tissues and, ultimately, metastasis.

Our interest in this field is defined by three key considerations. First, we are concerned mainly with ion channels that are gated by membrane *voltage*, i.e. *V*_m_ (due to its potential huge impact on cellular functioning, as indicated above). Other types of ion channel, including ligand-gated and mechanosensitive ion channels, also expressed in cancer cells, are not considered here [15, 16]. Second, we are limited, at least initially, to *carcinomas*, the major cancer type, derived from epithelial cells. Expression of voltage-gated ion channels may be expected not to be so prominent in such cells under normal conditions due to their classic “non-excitable” nature unlike, say, tumours of the brain where neuronal electrical activity is implicit. Thus, with carcinomas, we can operate from a “clean” baseline for the most direct interpretation of electrophysiological data. Other cancers (e.g. gliomas) are mentioned when deemed interesting. Third, we focus on *metastasis* since this is the main cause of death from cancer. In evaluating the available evidence, it is important also to be precise about the functional assays under consideration. For example, *in vitro*, the term “motility” is sometimes used to denote “free” movement of cells around the culture dish. There is also cell movement in scratch assays and across micropore-transwell filters, which we would term “lateral motility” and “transverse migration”, respectively [17]. On the other hand, “invasion” assays additionally involve proteolysis of the Matrigel coating of the transwell filters. This could be deemed an *in vitro* model of metastasis as it involves both proteolysis of Matrigel (which mimics the basement membrane) and transverse migration through micro-pores resembling intra/extravasation. Finally, directional movement in electric fields is called “galvanotaxis” (or “electrotaxis”) [18]. The latter is worthy of notice as epithelial tissues are intrinsically polarised with trans-cellular potentials.

## Functional expression and pathophysiology of VGSCs in metastatic carcinomas

At the heart of the cancer cell electrical excitability is the expression of functional VGSCs. Such expression was first reported for rat prostate cancer (Mat-LyLu) cells of strong metastatic potential [12, 19]. Weakly/non-metastatic isogenic AT-2 cells were devoid of such channels. Subsequently, a wide range of other carcinomas of human origin have been shown to express functional VGSCs *in vitro* and *in vivo* for reviews: [20–22].

Interestingly, of the 9 functional members of the VGSC multi-gene family, 2 subtypes appeared the most common—Na_v_1.5 (*SCN5A*) and Na_v_1.7 (*SCN9A*), each a different member of the VGSC molecular/pharmacological subfamily: tetrodotoxin (TTX)-resistant and TTX-sensitive, respectively [23]. Furthermore, where studied, the VGSC was found to occur as an embryonic splice variant, consistent with the channel expression being “oncofoetal”. This phenomenon has been demonstrated most clearly for Na_v_1.5 expression in breast and colon cancer [11, 24, 25]. The *de novo* generation of “neonatal” Na_v_1.5 (nNa_v_1.5) is probably a part of the “dedifferentiation” process in cancer and comes about by the alternate splicing of exon 6 giving rise to a channel variant that includes a unique, highly antigenic set of 7 amino acids in an extracellular region of the protein. Importantly, nNa_v_1.5 is pharmacologically distinguishable from the adult form of Na_v_1.5 (aNa_v_1.5) which is expressed mainly in the heart [26]. Furthermore, nNa_v_1.5 has a highly restricted expression pattern and appears mostly absent in normal tissues [27]. Thus, nNa_v_1.5 is a potential neoantigen. Na_v_1.7 in prostate cancer is spliced similarly but this generates only 1 amino acid difference [28]. The splicing has a significant effect on the conserved aspartate residue at position 211 in both channels. This switches to a lysine (i.e. a double charge change) in nNa_v_1.5 and is responsible for generating some of the key electrophysiological and pharmacological differences between nNa_v_1.5 and aNa_v_1.5 [26, 29]. Another important property of the cancer VGSC, again most clearly demonstrated for (n)Na_v_1.5, is the ability to generate a “persistent current” (*I*_NaP_) which is promoted by hypoxia, well known to develop in growing tumours [30]. This persistent (sometimes called “late”) current is also likely to contribute to the high level of sodium detected in cancer cells and patient tumours [31].

Over the years, evidence has grown steadily for a pro-metastatic role of the functional VGSC expression in line with the CELEX model [21]. The available evidence ranges from *in vitro* to man and shows that upregulation is early and occurs at a hierarchy of levels from mRNA to functional expression in several carcinomas [21]. Furthermore, both gene silencing and local or systemic pharmacological inhibition of the VGSCs (including with TTX, a highly specific natural toxin) have been shown to suppress, even eliminate, invasiveness *in vitro* and metastasis *in vivo* [21, 32–35]. This could occur without a significant effect on primary tumorigenesis, consistent with the notion that primary tumorigenesis (proliferation) and secondary tumorigenesis (invasion, metastasis) are controlled differently, even at least partially independently [e.g. 36, 37].

The reverse has also been demonstrated. Thus, Bennett et al. (2004) have shown that a non-invasive human prostate cancer (LNCaP) cell can be made invasive just by being transfected with a VGSC. In fact, these workers stated strongly that VGSC expression was “necessary and sufficient” for the cells to become invasive [38].

Finally, *I*_NaP_ can be blocked selectively by the anti-angina drug ranolazine which also inhibits invasiveness *in vitro* and full-blown metastasis *in vivo* [25, 32, 34, 35]. Importantly, a 10-year retrospective data mining study—real-world data (RWD)—revealed that breast, prostate and colon cancer patients taking ranolazine (for some other indication) had significantly reduced risk of dying from cancer (hazard ratio = 0.41) [39]. Even those cancer patients who started taking ranolazine some 4 years after diagnosis still benefited significantly (hazard ratio = 0.54).

In conclusion, substantial *in vitro* and *in vivo* evidence from a range of cancers and RWD from humans suggests unequivocally that VGSC activity promotes, in fact may even initiate, the metastatic process.

## Voltage-gated potassium channels

In the context of the CELEX model, the evidence for the involvement of functional VGPCs in cancers is rather mixed for at least two reasons. First, VGPCs are extremely diverse comprising some 40 genes in humans and representing the largest group of ion channels with wide-ranging kinetics [40]. Second, these channels are intimately associated with generation of the *V*_m_ and thus can bring about significant, diverse knock-on effects (e.g. on Ca^2+^ signalling, pH). In turn, such secondary effects can impact profoundly and differentially on cancer cell behaviours. For example, in one study, overexpression of K^+^ channels (K_v_1.5 and Kir2.1) *increased* invasiveness/metastasis but, as indicated above, this was due to *V*_m_ hyperpolarization and induced upregulation of cadherin-11 and MAPK signalling [41]. Furthermore, K^+^ channels may also be involved in glucose metabolism and glycosylation in cancer cells, but this aspect is outside the scope of the current perspective [42].

According to the CELEX model, the role of the VGPCs depends on the stage of the cancer. Early on (during primary tumorigenesis), VGPC activity would be elevated, driving proliferative activity, as it is well known to do [43]. As metastasis gets going, however, VGPC expression would be downregulated so as to promote the arising VGSC activity. Unfortunately, studies on the precise stage dependency of VGPC expression in carcinomas are lacking. Also, such a study would need to focus on the particular VGPC(s) involved in a given cancer and this is not always known. Even if known, the specificity of the available relevant antibodies can be questionable. Nevertheless, there is considerable evidence for the predicted stage-dependent involvement of K^+^ channels in cancer. For example, Fraser et al. [44] showed for *isogenic* rat prostate cancer cells (i) that K_v_1.3 was the dominant VGPC and (ii) that VGPC / K_v_1.3 inhibitors were much more effective in suppressing the proliferation of the weakly/non-metastatic AT-2 cells compared with the strongly metastatic, VGSC-expressing Mat-LyLu cells. An analogous result was obtained by transfecting the intermediate conductance calcium-activated potassium channel (IK) into the highly metastatic breast cancer cell line MDA-MB-231 in comparison with the spontaneously immortalised “normal” breast epithelial MCF-10A cells. The IK over-expression increased primary tumour growth and metastasis of MDA-MB-231 in orthotopic xenografts but, in contrast, the same treatment *decreased* MCF-10A proliferation and invasion [45]. The IK channel itself is voltage-independent but both the channel current and the associated Ca^2+^ are voltage-dependent, so the differential effect of the transfections could be due to the secondary impact of the *V*_m_ in these two contrasting cell lines.

Another corollary of the CELEX model is whether an agent that suppresses cancer progression through VGSC inhibition could, simultaneously, promote VGPC activity. Such an effect has been reported for local anaesthetics [46].

Most studies on VGPC expression in cancers to date have focused on K_v_1.3, K_v_10.1 and K_v_11.1 [47, 48]. These are discussed individually in the following sections.

### K_v_1.3

The case of K_v_1.3 (“delayed rectifier”) channels is easiest to assess since these channels activate by simple depolarization of *V*_m_ and then inactivate over 10 s of milliseconds, i.e. their kinetics overlap with VGSCs, as in classic action potentials [46]. Furthermore, as the so-called “lymphocyte channel”, K_v_1.3 has been extensively studied elsewhere as well, so good antibodies and a natural toxin (margatoxin) exist. Abdul and Hoosein [49] found a significant inverse correlation between K_v_1.3 protein expression and tumour grade in human prostate biopsies, just as the CELEX model would predict, and suggested that reduced K_v_1.3 expression could be associated with poor patient outcome. Similarly, compared with normal breast tissue, K_v_1.3 gene expression was decreased (via methylation) in human poorly differentiated breast carcinomas and younger patients [50]. In a more recent study on Mat-LyLu cells, margatoxin *increased* invasiveness [51]. Also, treatment of analogous human strongly metastatic breast cancer MDA-MB-231 cells with the general VGPC blocker 4-AP produced a similar effect and the increased invasiveness was suppressed by TTX [51].

The effects of some other Kv’s have also been studied. Although brain tumours are not central to the current review, K_v_1.5 expression was found to be higher in high-grade vs. diffuse astrocytoma [52] and lowest in glioblastoma [53]. Furthermore, there was a tendency with glioblastoma patients with high levels of K_v_1.5 expression to survive better [52]. In seminoma, *KCNC1* (K_v_3.1) was hypermethylated which would inhibit its expression, and this was associated with poor overall survival in patients [54]. Such results directly support the CELEX model.

### K_v_10.1

This channel, also a “delayed rectifier”, has the characteristic property of strong dependence on basal *V*_m_ for activation [48, 55]. Thus, channel activity can bring about significant hyperpolarization of *V*_m_ which could lead to a range of downstream effects including influx of Ca^2+^ and modulation of VGSC activity. There is evidence that expression of K_v_10.1 is restricted to parts of the brain and absent from normal epithelial tissues [56]. However, significant upregulation occurs in a wide range of *primary* carcinomas, possibly to promote proliferative activity [56]. On the other hand, the gene for K_v_10.1 (*KCNH1*) was found to be heavily methylated in human gastric cancer tissues [57]. In this state, expression may be expected to be downregulated, as for K_v_1.3 expression in breast cancer [50]. Functionally, there is substantial evidence that K_v_10.1 activity generally promotes cell cycle control and cell proliferation, migration, angiogenesis and resistance to hypoxia of cancerous cells [43, 58]. Such cellular behaviours can indirectly contribute to metastasis. Interestingly, the functional role of K_v_10.1 activity appears to be different in strongly vs. weakly/non-metastatic cells. Thus, in human breast cancer cell lines, K_v_10.1 channel activity enhanced proliferation of the non-invasive MCF-7 cells which do not express any functional VGSC [11, 58, 59]. In contrast, proliferative activity of the invasive/metastatic, VGSC-expressing MDA-MB-231 cells was not affected. These results agree with the observations involving K_v_1.3 on rat prostate cancer cells and are in line with the CELEX model [44]. The potential role of K_v_10.1 in tumour cell invasiveness and metastasis *per se* is not well understood. Hammadi et al. [60] and Valdés-Abadía et al. [61] showed for the MDA-MB-231 cells that downregulating K_v_10.1 activity (by siRNA or astemizole) slowed down lateral motility. Importantly, however, this effect could be an indirect consequence of the observed concomitant depolarization of *V*_m_ and reduced Ca^2+^ influx [60]. In a clinical study, expression of K_v_10.1 in brain metastases relative to matched primary carcinomas (various subtypes) was found to be highly variable—higher in 60%, unchanged in 26.7%, and lower in 13.3% of cases (*n* = 30) [62]. It is possible that it is not only how much channel is present that is important, but also if it is (still) cell cycle-dependent. In fact, most of the effects seen with K_v_10.1 could be due to extemporary activity rather than channel functioning in absolute terms (L. Pardo, personal communication).

Clearly, more work is required to elucidate directly the mechanistic role of K_v_10.1 in cancer cell invasiveness and metastasis. It will be interesting, for example, to determine the pathophysiological consequence(s) of the *KCNH1* methylation in human gastric cancer which is known to express Na_v_1.7 at least at mRNA level [63].

### K_v_11.1

The case for K_v_11.1 is even more complex since the channel can exist in two different functional states [64, 65]. In the *non-conducting* mode, K_v_11.1 associates physically with integrin-β1 and promotes cellular behaviours involved in the cancer process. This appears to be its primary mode of action in cancer. In contrast, when the integrin association is disrupted, the channel functions in a *conducting* mode and its impact on metastasis *in vivo* is reduced [64]. Consistent with the latter, promoting K_v_11.1 activity pharmacologically in strongly metastatic breast cancer MDA-MB-231 cells *in vivo* suppressed metastasis in part by reprogramming, in fact reversing, epithelial-mesenchymal transition [66, 67]. A recent study on canine mammary tumours found (i) that K_v_11.1 protein expression was significantly higher in grade I and II tumours than in grade III tumours and (ii) that there was a trend for disease-free and overall survival to be positively correlated with K_v_11.1 expression [68]. These effects also agree with the CELEX model.

Taken together, the available evidence for VGPC expression in cancer cells is broadly supportive of the CELEX model. However, further work is required to verify that the proposed pathophysiological role of given VGPCs depends on the stage of the cancer process. Such a situation would be in line with the increasing evidence for cancer cell plasticity during malignant progression [69].

## Mechanistic consequences: action potentials in *cancer* cells

The CELEX model would predict that strongly metastatic carcinoma cells specifically should generate action potentials (APs). The evidence for this is discussed in the following sections.

### *In vitro* evidence

**Fig. 2:**
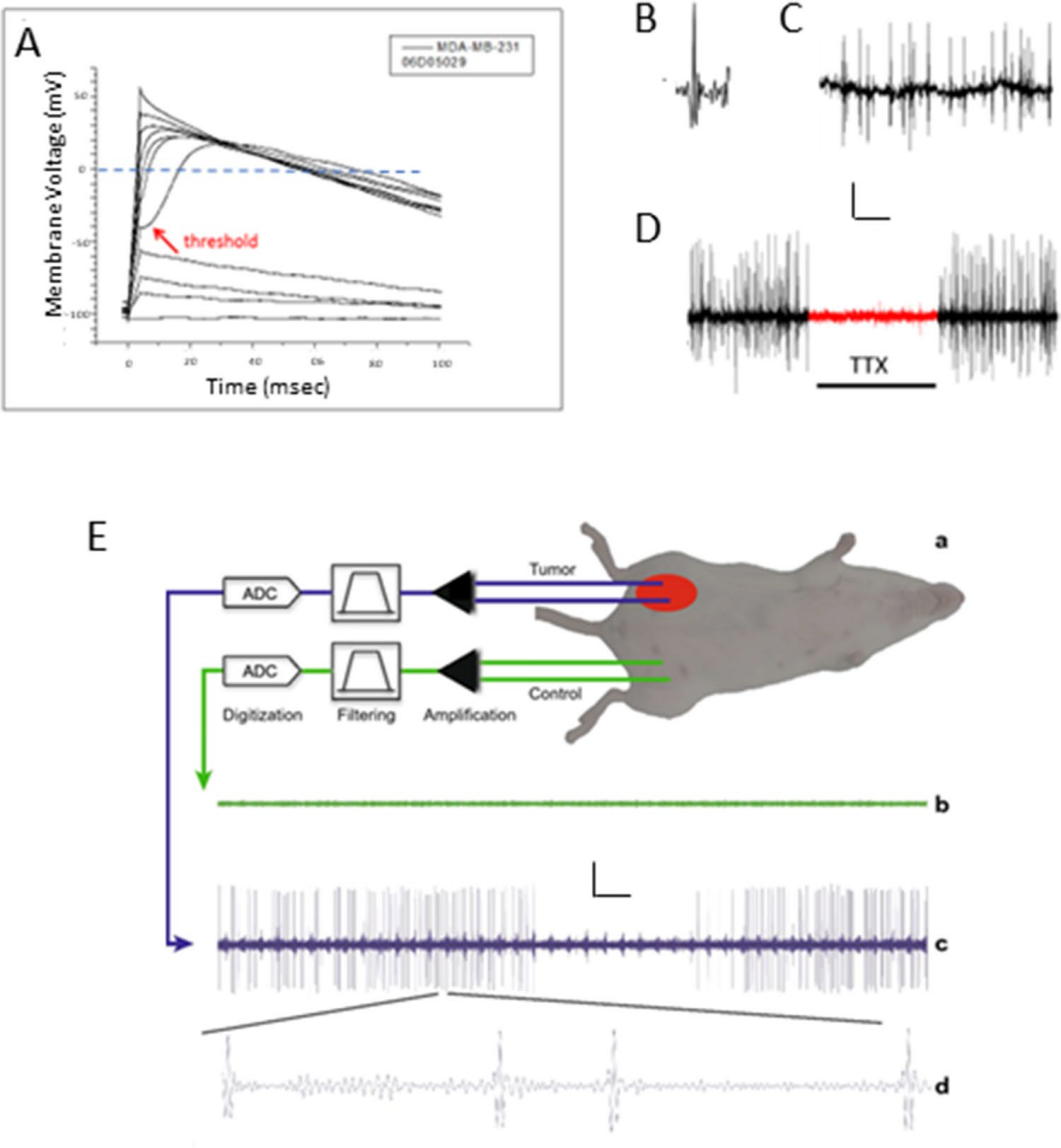
Action potential activity recorded from breast tumours. **A**–**D** Data from human MDA-MB-231 cells recorded in different configurations. **A** Current clamp recording showing “all-or-none” type responses once a threshold is passed (indicated by the arrow). The cell was stimulated from the holding level (− 100 mV) by currents in the range 0 to 340 pA (20 pA steps, 2 s intervals). Only every other trace is shown for clarity. The dashed horizontal bar marks the 0 mV level to emphasise the overshoot. Note that the action potentials appear broad probably due to the cells having little outward currents for repolarisation. Recording by Rüstem Őnkal. Modified from Djamgoz [10]. **B**–**D** Recordings from micro-electrode arrays. **B** An individual spike. **C** Train of spikes. **D** Complete and reversible blockage of the spiking activity by application of 20 μM TTX. Scale bars: 20 pA/210 ms (**B**), 30 pA/2 s (**C**), 25 pA/8 min (**D**). Modified from Riberio et al. [72]. **E** Chronic recordings from mice bearing 4T1 breast tumours. (a) Two microwire electrodes (each 125 µm diameter) implanted in the mammary tumor mass revealed a high level of electrical activity with classic spike waveform (c,d). The other two “control” microwires on the contralateral side detected no activity (b). Scale bars: 15 μV/1 s (c), 15 μV/10 ms (d). Modified from McCallum et al. [73]

Although several types of carcinoma cell lines have been shown repeatedly to express VGSC activity (“membrane current”) under voltage-clamp recording conditions, whether these cells are capable of generating APs has not been studied as much. Blandino et al. [70] originally showed that APs blocked by TTX could be elicited in cells of human small-cell lung cancer, which is an aggressive tumour. Djamgoz [9, 10] provided further evidence showing that the human strongly metastatic breast cancer (MDA-MB-231) cells are also capable of generating APs under current-clamp recording conditions (Fig. 2A). In both cases, however, the membrane potential had to be artificially hyperpolarized before APs could be elicited. Indeed, there was a theoretical objection to the functionality of the VGSCs since at the rather depolarized (− 20 to − 30 mV) resting membrane potentials of cancer cells, the channels would be expected to be mainly inactivated. In turn, this seemed at odds with the fact that TTX (i) is well known to block VGSCs by binding to the selectivity filter within the pore region, i.e. when the channel is in the open state and (ii) binding inhibits metastatic cell behaviours. In fact, we now know from less invasive voltage-sensitive dye recordings that the resting *V*_m_ of cancer cells is not static but undergoes transient hyperpolarizations enabling the VGSCs to spend a significant time in an open state [71]. On the other hand, in current-clamp experiments, *V*_m_ needs to be hyperpolarized artificially in order to promote the activation of the VGSCs.

More recently, a novel, non-invasive electrophysiological recording technique has been applied to determine whether human cancer cells of strong metastatic potential would indeed generate VGSC-dependent spontaneous activity [72]. Thus, plating MDA-MB-231 cells onto micro-electrode arrays (MEAs) employing gold electrodes showed clearly (i) that the cell ensembles were spontaneously active with “spikes” and (ii) that the activity was blocked reversibly by TTX, just as predicted from many years of pharmacological experiments on individual cells (Fig. 2B–D). A similar earlier study also found spiking activity in analogous human prostate cancer PC-3 cells “at rest” [74]. The activity was thought to be mainly Ca^2+^-dependent since Gd^3+^ blocked the spikes. However, the concentration of Gd^3+^ used (250 μM) could readily affect VGSC activity [75]. As noted above (Sect. 2), rat and human prostate cancer cells also express a functional VGSC and this is likely to be the TTX-sensitive subtype Na_v_1.7 [28, 76]. In a recent preliminary study, extracellular spikes were recorded from several human triple-negative breast cancer cell lines with the spike frequency increasing (up to ca. 3.5 kHz) in line with the cells’ metastatic potential (10.1101/2024.03.16.585162).

In conclusion, resting carcinoma cells are indeed capable of spontaneously generating Hodgkin-Huxley type APs as defined by (i) the existence of a threshold for excitation, (ii) the all-or-non-nature and (iii) complete and reversible blockage by TTX.

### *In vivo* evidence

We would expect this question to be answered definitively in the foreseeable future since the relevant technology (e.g. electrophysiological recording from tumour slices) has already been tried and successfully verified [77]. In one such application, Simon et al. [78] recorded electrophysiological activity in slices of the brain implanted with breast tumours derived from MDA-MB-231 cells, aiming to mimic metastasis. Although clear spikes were recorded around the lesions, the cellular origin(s) of the activity was not determined. In a more definitive whole-animal *in vivo* study, McCallum et al. [73] demonstrated that breast tumours induced by inoculating the “triple-negative” 4T1 cells in a mouse model were spontaneously active with APs (Fig. 2E). Non-tumour areas did not show any such activity. We should note, however, that since tumours possess active nerve input, it will be necessary in such recordings to separate the intrinsic electrical activity of the tumour from the nerve activity.

In conclusion, APs occur in tumours *in vivo* as *in vitro*. Such studies can enhance our understanding of the dynamic contribution of VGSC activity to metastasis by determining for example (i) the correlation between any electrical activity and the cells’ metastatic behaviour and (ii) whether the presumed pre-metastatic spike activity would be maintained in secondary tumours or it switches off once the target is reached.

### Possible functional role of spiking activity in *cancer* cells

Here, we can learn a lot from neuroscience which has shown that VGSC/AP activity can sub-serve a wide range of cellular functions from the most fundamental (e.g. gene expression) to whole-cell behaviour (e.g. motility, secretion) to multicellular (e.g. network interactions and patterning). Recent work is suggesting increasingly that cancer cells are in two-way communication with several cell types including epithelial cells, fibroblasts, endothelial cells and immune cells within the tumour microenvironment (TME) [79]. Cancer cells themselves form elaborate networks by extending “tunnelling nanotubes” (TNTs), similar to neurites, which can be 500 µm or longer [80]. This distance is comparable to the separation of Nodes of Ranvier in myelinated nerve fibres (200 µm–2 mm), consistent with effective conduction of APs. Thus, generation of APs would be highly expedient for signal transmission along TNTs and enable extensive cellular communication within the TME [81].

Also, intriguingly, tumours are innervated. On the one hand, it is well known that cancer cells use the nerve connection as a physical conduit to migrate out of the tumour mass [82]. In fact, the cancer cell-nerve terminal connection is *two-way* [83, 84]. Thus, cancer cells can use APs to feed back onto nerves or other cells within the tumour microenvironment. A major input to tumours is provided by the vagus nerve, the largest of the cranial nerves and a prominent component of the peripheral nervous system, which innervates many of the body’s essential organs [85]. Importantly, the innervation is an integral part of the cancer process and can have a significant impact on prognosis. For the nerve input to carcinomas, our best understanding comes from human prostate cancer where the sympathetic (adrenergic) input contributes to early tumourigenesis (proliferation) whilst parasympathetic (cholinergic) activity promotes later stages, e.g. invasion [79]. Input from the spinal cord promotes prostate cancer and, consistent with this, men with spinal cord injuries, compared with age-matched healthy individuals, had 33% lower risk of developing prostate cancer [86]. Importantly, the nerve-cancer cell “synapse” involves positive feedback so the active nerve input to the cancer cells can lead to secretion of growth factors from the latter which can induce further neurogenesis and angiogenesis [84, 87]. This feedback appears to involve release of cancer-derived exosomes [88]. Such interactions are likely to involve APs in cancer cells, at least in part, especially as tumours grow to a pro-angiogenic size of 0.5 mm or bigger.

## Pathophysiological role of sodium in *cancer* cells: an overview

Another consequence of the AP activity in cancer cells is rise in intracellular sodium concentration, especially under hypoxic conditions, accompanying tumour growth, when the VGSC develops a “persistent current” [30]. The increased sodium content of tumours is detectable by clinical ^23^Na-MRI [89]. As a result, the trans-membrane inward sodium gradient is weakened, and this impairs sodium-dependent transport mechanisms such as for amino acids and glucose [31]. The VGSC also promotes extracellular acidification, through mechanisms like sodium-hydrogen exchange (NHE1) and sodium-bicarbonate transport, with important consequences for the metastatic process. We should note, however, that from the impact of the VGSC-driven Na^+^ influx on NHE1, one would expect *reduced* H^+^ extrusion, the opposite of what is known to happen. There are two possibilities: (i) The VGSC-NHE1 interaction is allosteric/protein–protein (rather than an ionic effect). This has already been proposed [90]. (ii) There is an intermediary mechanism (currently unknown) between the VGSC-driven Na^+^ influx and NHE1 which results in increased exchanger activity and the observed pericellular acidification. As regards the consequences of the acidification, first, proteolytic enzyme activity increases, and this promotes the invasion of the surrounding tissues, leading ultimately to intravasation [91]. Second, as shown more recently for several carcinoma cell lines, the acidic environment deters the infiltration of the tumour by CD8 + T-lymphocytes and immune response is curtailed [92]. Although intracellular calcium and related signalling also depends strongly on the sodium gradient, this aspect has not yet been studied in detail.

## Clinical implications of the CELEX model

The two ionic components of the CELEX model (VGSC and VGPC) individually offer much clinical potential in terms of both diagnosis and therapy and these have been discussed extensively elsewhere [93–96]. In terms strictly of the model overall, two additional possibilities can be considered, as follows.

### Electrodiagnosis

A clinically viable cancer marker must satisfy a number of important criteria including (i) expression early in the cancer/metastatic process; (ii) minimally invasive detection, e.g. not involving excisional biopsy and (iii) being functional “companion diagnostic” i.e. enabling matching therapy. Detection of APs in tumours would meet all these criteria. The AP activity recorded by McCallum et al. [73] *in vivo* peaked at two timepoints (on days 15.9 and 20.0) the latter corresponding statistically to the detection of metastasis in lungs. In cancer patients, this approach would be most directly applicable to superficial tumours like melanoma but may also be possible for deeper tumours. Furthermore, flexible MEAs can be employed *in situ* for better contact [97]. Thus, clinical measurements of electrical (AP) activity from suspected tumour regions of the body could give rise to the idea of an *electro-oncogram*, analogous to the electrocardiogram (ECG) and electroencephalogram (EEG). However, there could be a signal–noise problem in such applications since the density of the VGSC current in cancer cells (few 100s pA), at least *in vitro*, is noticeably smaller than, say, in central neurons or cardiomyocytes (> > 1 nA). Nevertheless, technologies with improved signal-to-noise ratio could overcome this limitation. More broadly, electrical impedance tomography (EIT) of tissues of cancer patients is already proving clinically viable *ex vivo* and *in vivo*. This has been shown for breast cancer [e.g. [98, 99] and ovarian cancer [100]. Another possibility would be to follow *V*_m_/AP activity *in vivo* using fast genetically encoded voltage indicators (GEVIs), now used routinely (e.g. in combination with viral transduction) to study mammalian brain activity *in vivo* [101–103]. Overall, the current evidence suggests strongly that occurrence of VGSC/AP activity in a tumour will lead to metastasis. Thus, detection of such activity non-invasively can greatly facilitate the therapeutic decision-making process including whether surgery would be necessary or not and, if so, how extensive this should be. Furthermore, with the rapid advances being made in material sciences, it could be possible to monitor APs with wearable devices and thus continuously monitor possible disease state and response to treatment [104].

### Electroceutical therapy

Bioelectricity is being applied in cancer therapy in a number of different ways. These include “tumour treating fields” (TTFs) which are approved by the FDA for use against gliomas, and other applications may follow [105]. Another clinically established technique is “electroporation” in which nanosecond pulses of voltage are used to facilitate penetration of chemotherapeutic agents or genes into tumours [106]. More recently, Ju et al. developed a wearable biological patch (“eT-patch”) incorporating a photothermal ionic-gel potentiated by electrostimulation for treating skin cancer under real-time visual examination [107]. Application of the technique over 15 days to mice inoculated with melanoma resulted in an almost tenfold reduction in tumour growth and significantly increased survival.

Of course, any clinically intended manipulation of the cancer VGSC and/or VGPC should not impact adversely on other channels expressed elsewhere in the body. First, fortunately, the VGSC does have two independent properties that are intimately related to the cancer/metastatic process: (i) expression as a neonatal splice variant and (ii) generation of hypoxia-driven *I*_NaP_. These can be targeted selectively using respectively a monoclonal antibody (Duranti C. et al. – in preparation) and ranolazine [108]. The case for VGPC “openers” is trickier since there are few such agents and what there is remains to be fully characterised. Nevertheless, the possibility of targeting solid tumours with potassium channel activators was suggested even a decade ago by Trechot [109]. Interestingly, there is one “neuronal” VGPC, K_v_7, for which an “opener”, retigabine, has been approved by the FDA as an anticonvulsant. Matthews et al. [110] tested the effects of retigabine on a rodent glioma-neuroblastoma hybrid (NG108-15) cell line and found that it significantly *inhibited* proliferation. Further work on human glioma U87 cells showed (i) that NS1643, an activator of K_v_7 and hERG channels, also inhibited proliferation and (ii) that NS1643 and retigabine potentiated the anti-proliferative effect of temozolomide which is used clinically as an anti-glioma drug. It would be worthwhile to extend these studies to determining possible effects of retigabine on invasiveness and to develop openers to other K_v_’s more commonly expressed in carcinomas, e.g. K_v_1.3, K_v_10.1 and K_v_11.1 (Sect. 3).

**Fig. 3:**
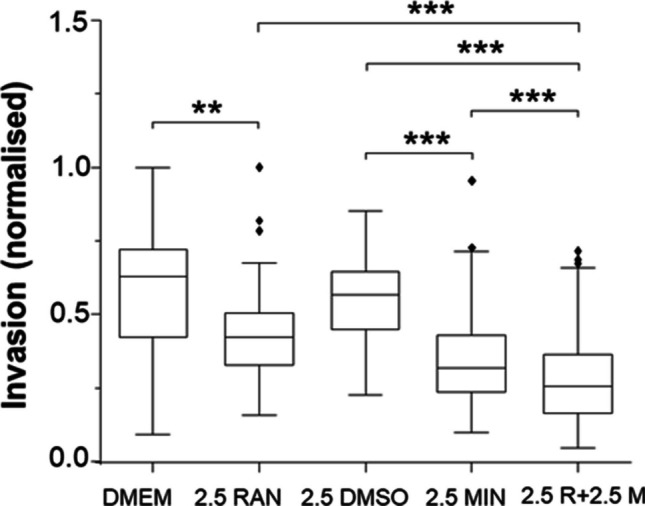
Effects of ranolazine, minoxidil and their combination on invasiveness of MDA-MB-231 cells. Box plots showing the effects of 2.5 µM ranolazine (RAN), 2.5 µM minoxidil (MIN), and their combination treatments on Matrigel invasiveness under hypoxia. DMEM and DMSO denote the respective control media. Cellular invasiveness was normalised to the largest number of invaded cells viewed per insert. The box plots show medians, interquartile ranges, 5% and 95% confidence intervals and outliers. Asterisks indicate statistical significance as *P* < 0.01 (**) and *P* < 0.001 (***). Data redrawn from Qiu et al. [111]

In terms of the CELEX model per se, we would predict that combining a VGSC blocker with a VGPC opener could provide an additionally effective anti-metastatic strategy. This has been tested recently in an *in vitro* study by Qiu et al. [111]. Since the field of specific VGPC openers is still emerging, minoxidil was used as a well-understood opener of ATP-gated/closed potassium (*K*_ATP_) channels. Combination of minoxidil with ranolazine produced a significant (ca. 40%) additive effect in suppressing the invasiveness of human breast cancer (MDA-MB-231 and MDA-MB-468) cells (Fig. 3). In fact, in the presence of minoxidil, ranolazine proved effective at concentrations some tenfold less than the current clinical dosage. Replication of this result in the clinic would be greatly beneficial as regards both treatment efficacy and reduced side effects [108].

## Concluding remarks

The “bioelectricity of cancer” is an integral part of the burgeoning field of *cancer neuroscience* but has a surprisingly much longer history. Studies of EIT started in the 1940s with cervical cancer [112]. The idea of human cancers being innervated was discussed even earlier [113, 114]. However, it is only now that such observations are gaining mechanistic insights. The CELEX model strives to do this for metastasis. Following the exciting discovery of voltage-gated ion channels in carcinomas, different groups focused on different channels expressed in different cancers. In fact, the reality is likely to be that “all major ion channels are expressed in all cancers”, with only subtypes and intrinsic regulatory mechanisms differing. This would be like the brain. We can imagine the cancer process, therefore, as an orchestra with different musical instruments representing different channels. The expression/activity of given channels will be dynamic depending, in the first instance, on the stage of the cancer. Again, this is like an orchestra with different instruments contributing to the different stages of the music! In order to appreciate the cancer process from a bioelectric point of view, therefore, it is imperative, that we start by bringing together the different ion channels (and their individual interactive partners). The natural starting combination is VGSC + VGPC, just as in the most basic bioelectric signal of the body—the action potential.

The essential components of the CELEX model are evidence-based and are increasingly being supported by new experiments such as the MEA, *in vivo* recordings and evidence from humans. Nevertheless, like any other model, it will need to be tested more thoroughly and, if necessary, modified accordingly whilst its clinical potential is realised. Regarding the latter, the possibility of managing systemic metastatic disease non-toxically and cost-effectively using ion channel modulators and their appropriate combinations, as indicated by the CELEX model, is exciting and should be welcomed by cancer patients.

## Data Availability

Data sharing is not applicable to this article as no datasets were generated or analysed in the study.

## Abbreviations

AP: Action potential
EIT: Electrical impedance tomography
*I*_NaP_: Voltage-gated sodium channel persistent current
K_v_1.3: Voltage-gated potassium channel subtype 1.3
K_v_10.1: Voltage-gated potassium channel subtype 10.1
K_v_11.1: Voltage-gated potassium channel subtype 11.1
MEA: Microelectrode array
Na_v_1.5: Voltage-gated sodium channel subtype 1.5
nNa_v_1.5: Neonatal splice variant of voltage-gated sodium channel subtype 1.5
Na_v_1.7: Voltage-gated sodium channel subtype 1.7
RWD: Real-world data
TME: Tumour microenvironment
TNT: Tunnelling nanotubes
TTX: Tetrodotoxin
VGPC: Voltage-gated potassium channel
VGSC: Voltage-gated sodium channel
*V*_m_: Membrane potential
